# Mechanical Detection of the De Haas–van Alphen Effect in Graphene

**DOI:** 10.1021/acs.nanolett.2c02655

**Published:** 2022-12-13

**Authors:** Juuso Manninen, Antti Laitinen, Francesco Massel, Pertti Hakonen

**Affiliations:** ‡ Low Temperature Laboratory, Department of Applied Physics, Aalto University, PO Box 15100, AaltoFI-00076, Finland; § QTF Centre of Excellence, Department of Applied Physics, Aalto University, PO Box 15100, AaltoFI-00076, Finland; ⊥ Department of Physics, Harvard University, Cambridge, Massachusetts02138, United States; || Department of Physics, Nanoscience Center, University of Jyväskylä, JyväskyläFIN 40014, Finland; ○ Department of Science and Industry Systems, University of South-Eastern Norway, PO Box 235, Kongsberg3616, Norway

**Keywords:** graphene, de Haas−van Alphen effect, Corbino geometry, nanomechanics

## Abstract

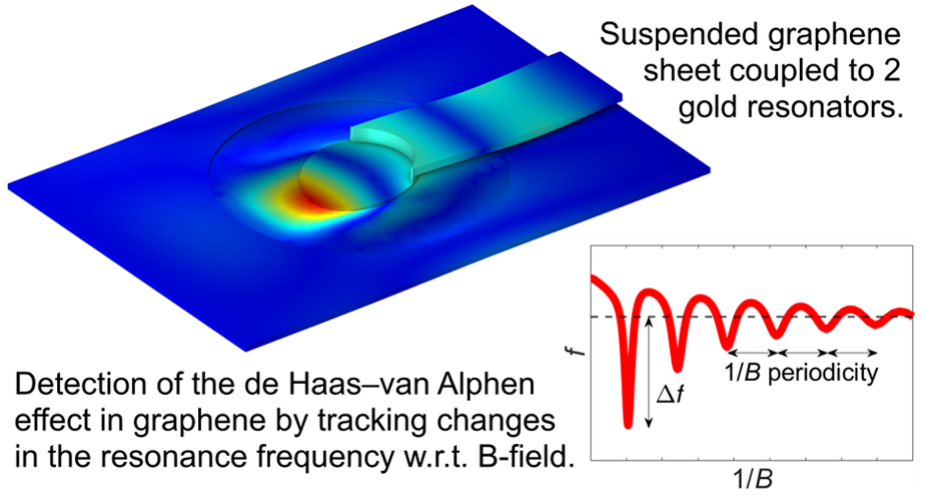

In our work, we study the dynamics of a graphene Corbino disk supported by a gold mechanical resonator in the presence of a magnetic field. We demonstrate here that our graphene/gold mechanical structure exhibits a nontrivial resonance frequency dependence on the applied magnetic field, showing how this feature is indicative of the de Haas–van Alphen effect in the graphene Corbino disk. Relying on the mechanical resonances of the Au structure, our detection scheme is essentially independent of the material considered and can be applied for dHvA measurements on any conducting 2D material. In particular, the scheme is expected to be an important tool in studies of centrosymmetric transition metal dichalcogenide (TMD) crystals, shedding new light on hidden magnetization and interaction effects.

As theoretically shown by Landau and Peierls in the 1930s,^1,2^ the de Haas–van Alphen (dHvA) effect consists of a periodic oscillation of the magnetization (and the magnetic susceptibility) as a function of the magnetic field. Along with other magnetic-field-induced phenomena, such as the Shubnikov–de Haas (SdH) conductance oscillations, the quantum Hall effect, and quantum capacitance oscillations, the origin of the dHvA effect is a consequence of the modification of the electronic spectrum in the presence of a magnetic field. Since, in this case, electronic motion becomes quantized due to the formation of Landau levels, which are ultimately responsible for the nontrivial properties of the considered electronic system, it is quite natural that the dHvA effect has served as the central probe in studies of the shape of the Fermi surface in normal metals.

Besides investigations of the dHvA effect in conventional three-dimensional (3D) materials, magnetic properties of two-dimensional (2D) materials have been investigated actively.^3,4^ Unlike the 3D case, where the field dependence of the magnetization is described by the classical 3D Landau–Kosevich formula, for 2D samples, the magnetization shows a characteristic sawtooth pattern both for massive and massless Dirac fermions.^5^

On the experimental side, in 2D, the dHvA effect was first observed by Eisenstein et al. in 1985^6^ in a 2D electron gas (2DEG), while a clear sawtooth pattern for the magnetization vs inverse magnetic field predicted in ref (2) was resolved about ten years later.^7^ For Dirac electrons comparatively fewer results have been obtained: SdH conductance oscillations have been reported^8−10^ in graphene, experiments have only recently revealed the dHvA effect.^11^

Focusing on detection techniques of magnetic properties based on mechanical motion, surface acoustic waves (SAW) have extensively been used for imaging of integer and fractional quantum Hall states (QH)^12^ in conventional GaAs 2DEG systems. In SAW-based techniques, the mechanical motion is coupled to the electron system due to piezoelectric response of GaAs, and variation in the compressibility of the electron system modulates attenuation and sound velocity in the material. Apart from SAW resonances, QH states in a 2DEG have also been investigated through curling,^13^ cantilever,^14,15^ and torsional modes.^6^

In our work, we report the dHvA measurement of graphene membrane in a Corbino geometry coupled to a gold (Au) mechanical resonator. The central idea of our technique consists in relating the shift of the mechanical resonant frequency of a graphene/Au structure to the oscillations of the magnetic susceptibility that characterize the dHvA effect. The idea of using mechanical motion to measure the magnetic properties of a graphene membrane introduced here can be considered as a part of the emerging field of sensing with 2D mechanical resonators.^16^

The investigation of the dHvA effect in suspended graphene membranes was first discussed in ref (17) where the frequency shifts of an all-graphene structure were analyzed both from a magnetization and a quantum capacitive perspective. The latter was then considered as the most natural description of the experimental results presented in ref (18). Similarly, ref (19) used the magnetic field dependence of the chemical potential (and the quantum capacitance) to explain observed frequency shifts without, however, explicitly relating the observed mechanical frequency shifts to the sample magnetization.

Our theoretical analysis shows how the description in terms of magnetization and chemical potential are two complementary interpretations of the same problem, furnished by the universal thermodynamic relation between quantum capacitance and magnetic susceptibility (see the Supporting Information). Furthermore, our experimental configuration with separated 2D sample and metallic resonator parts is an advancement of the device design employed in refs (18 and 19). The developed measurement setting allows the exploitation of the possibilities offered by suspended resonators in determining the magnetic properties of Dirac fermions in graphene and, in principle, the carrier-dependent magnetic behavior in other 2D materials, such transition metal dichalcogenides (TMDs) and 2D heterostructures.^21^ Furthermore, in graphene, our measurement setup is not limited to the observation of integer Hall states. In particular, we envision that the observation of fractional states is possible, along with the investigation of the interplay of electrical and mechanical degrees of freedom,^22^ for example, in the case of the formation of a Wigner crystal.^23^ Other possibilities include magnetization measurements of magic-angle twisted bilayer graphene^24^ and magnetization measurements of the emergent ferromagnetism in three-quarters filling twisted bilayer graphene.^25^

The sensitivity of our experiments is set by the frequency resolution of the resonance peak position, approximately 25 Hz. This frequency resolution corresponds to ∼10^4^ Bohr magnetons, which is 6 orders of magnitude better than in the torque magnetometer work of ref (7). Compared with the cantilever work of ref (14), our sensitivity is 2 orders of magnitude better. After optimization of the device parameters and improving the frequency resolution, similar sensitivity as in the work of Bleszynski-Jayich et al. can be obtained.^15^

Our approach is along the lines of cantilever sensing,^15^ but differs in the sense that our method, in principle, allows us for a streamlined investigation of different materials independent of the probing mechanical structure (in our case the Au structure), avoiding the issues related to glued/deposited cantilevers. The method is thus applicable to any 2D material, many of which can be fabricated into mechanical resonators.^26,27^

Our experiments were carried out on two devices: B2 (Figure 1b) and B1.5 (Figure 1c), additional images of the simulated mode shapes in the devices are shown in the Supporting Information. Device B2 consists of *two Au beams*, one graphene Corbino disk, and a back gate to which a voltage *V*_g_ is applied, controlling the charge density *n* on the graphene disk. The graphene Corbino disk couples the two Au beams together mechanically; the parallel Au beams are located at different heights, about 150 nm apart, supported by a bend in the center of the upper Au beam. Device B1.5 consists of *one and a half Au beams*: the top Au beam has been replaced by a gold cantilever. The specific choice of using gold (with a very thin layer of chromium) resonators stems from their low contact resistance to graphene and long-term stability.^29^ In contrast to more conventional geometries,^18,19^ the adopted Corbino disk geometry allows well-defined measurements based on pure σ_*xx*_ component, without problematic mixing of σ_*xx*_ and σ_*xy*_ components. Furthermore, Corbino geometry facilitates explorations over a wide range of Landau levels because of its capability of withstanding larger charge densities than devices with free edges. Transport via phonon-enhanced hopping conduction appears to be an additional useful characteristics that allows for detection of interaction-governed states in these disks (Wigner crystal^30^ and fractional quantum Hall states^31^).

**Figure 1:**
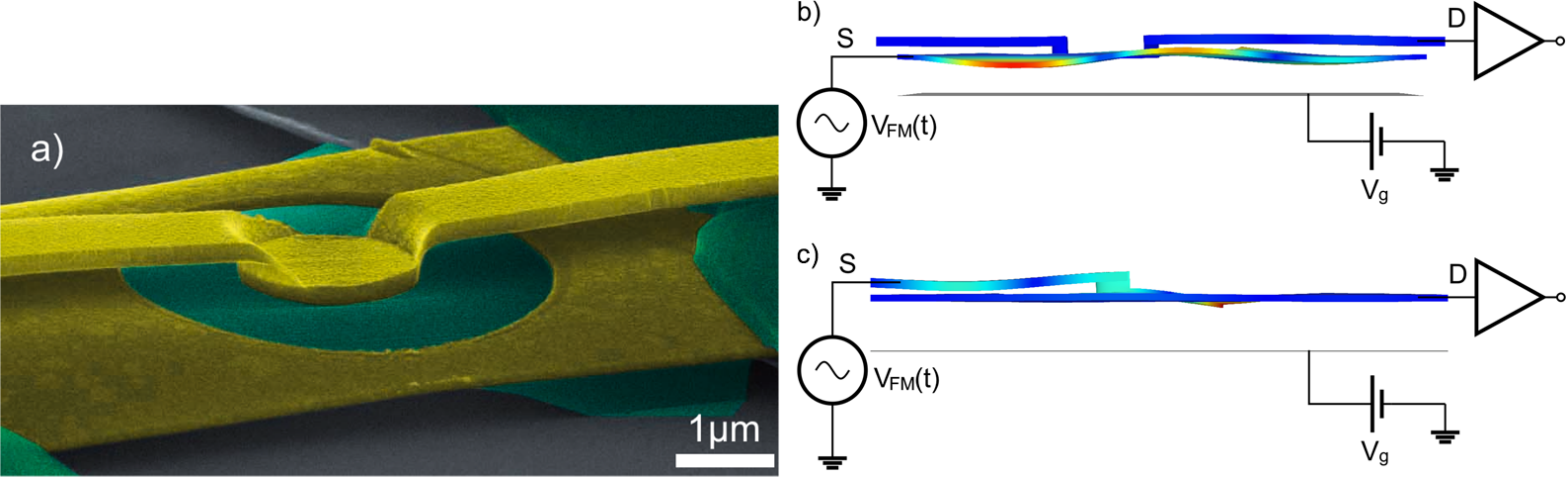
Sample structure and key mechanical modes. (a) SEM image of the measured device B2. The ring-shaped graphene colored green, Au parts appear as yellow, polymer support as dark green, and the substrate is gray. The length of the lower gold beam amounts 8 μm. Schematic of our measurement method for the device (b) B2 and (c) B1.5 consisting of two Au electrodes, one graphene Corbino disk, a back gate voltage *V*_g_, and the frequency modulation voltage *V*_FM_. The graphene/Au structure acts as a mixer between the voltage *V*_FM_(*t*) and the mechanical motion, allowing us to detect the mechanical motion through the measurement of the mixing current *I*_mix_(*t*). The mode shapes in b and c (not to scale) are obtained from FEM simulations of the respective devices utilized in this study are depicted with a color gradient highlighting the physical displacement.

The mechanical resonance properties of the samples were investigated using the FM mixing technique.^32,33^ In the FM technique, an FM source-drain voltage *V*_FM_(*t*) in Figure 1b, c is responsible for a source-drain current (mixing current *I*_mix_), which can be shown^32,33^ to be related to the amplitude and phase of the mechanical motion of the resonator at the modulation frequency ω_L_ as . Scanning through it, it is possible to reconstruct the position and line width of the mechanical resonance (see inlay panel of Figure 3). FM mixing was employed here due to the clear-cut form of the mixing signal, exhibiting a sharp and consistent three-lobed peak structure with sharp 180° phase flips, see the inlay panel of Figure 3.

Owing to the difference in effective mass between the Au and graphene portions of the devices, two basic types of resonances were observed: (low-frequency 10–40 MHz) combined gold-graphene modes and (high-frequency ≳90 MHz) pure graphene resonances in the Corbino disk.^34^ The low-frequency resonances (hereafter “Au modes”) are essentially governed by the gold structures, with the dynamics of the graphene membrane being dictated by the motion of the graphene/gold boundary conditions. For the latter, given the diamagnetic character of Au, the gold structure acts as a mechanical detector of the magnetic properties of the graphene disk. In addition, due to the mechanical properties of the Au beams, for the Au modes, there is a wider range of driving fields for which the linear detection of the quantum Hall states in graphene is possible in comparison with pure graphene modes. For these modes, the linear regime is limited to oscillation amplitudes around 100 pm.^35^ For these reasons, we focus here on the former ones.

The quality of the investigated graphene disks—exhibiting appreciable built-in strain, inferred from the unidirectional corrugations of the graphene disk parallel to the cantilever—was preliminarily assessed by measuring the device conductance *G*_d_(*V*_g_, *B*) (Landau fan diagram, Figure 2a). The degeneracy of the low-*B* QH states (ν = 2, 6, 10, ...) is lifted at fields *B* ≥ 0.5 T (see Figure 2a). For even stronger fields (*B* ≈ 3 T, not shown here), *G*_d_(*V*_g_, *B*) bears the signature of the fractional QH state ν = 1/3 (see refs (30 and 36)).

**Figure 2:**
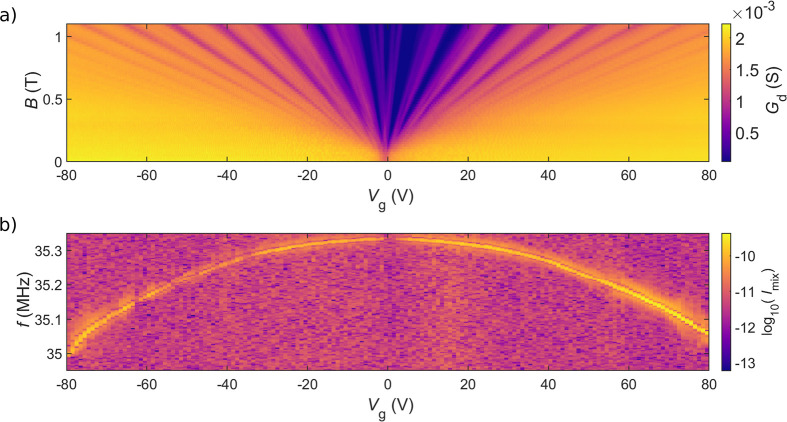
Landau fan diagram and capacitive softening. (a) *G*_d_(*V*_g_, *B*) (Landau fan diagram) as a function of the gate voltage *V*_g_ vs the magnetic field *B* plane, measured up to *V*_g_ = 80 V (*n* = 5.7 × 10^11^ cm^–2^). Above *B* ≃ 0.5 T, it is possible to observe the lifting of the Landau level degeneracy. (b) *V*_g_ dependence of the logarithm of mixing current log_10_(*I*_mix_) of the 35 MHz resonance for *B* = 0. The expected capacitive softening for the mechanical resonance is observed.^35^

At the mechanical resonance, both *G*_d_(*V*_g_, *B*) and the phase of the mixing current *I*_mix_ obtained through the FM technique were found to reflect the nontrivial *B* dependence of the electronic properties (see Figure 3: the local minima of *G* coincide with the upward phase flips in *I*_mix_ (see the Supporting Information). For this reason, the phase flips in *I*_mix_ can be employed as sensitive detectors of QH states in suspended graphene. In our case, a Landau level sequence up to ν = 30 can be resolved in Figure 3.

**Figure 3:**
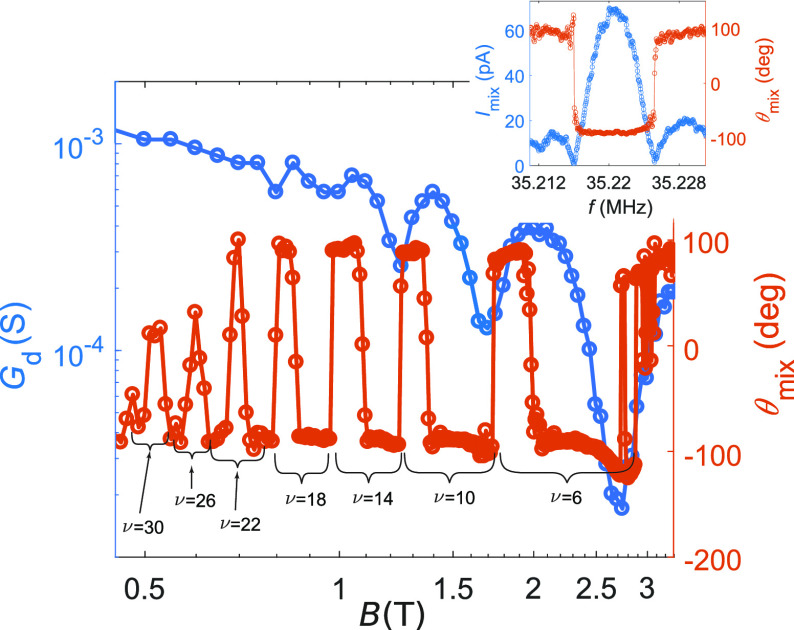
Conductance and the mixing current phase at resonance as a function of the magnetic field (*V*_g_ = 55 V). The upward phase flips correspond to the minima of *G*_d_(*V*_g_, *B*), providing a reliable signature of the transition from one Landau level to the next. Inlay panel: Mixing current *I*_mix_ and its phase for the 35 MHz resonance in zero magnetic field. The three-lobed structure of *I*_mix_ allows us to characterize with good accuracy the shift of the mechanical resonant frequency through the observation of the position of the two lateral dips. The location of the dips also corresponds to the phase flip of the mixing current, providing us with an alternative tool to characterize the frequency shifts.

Our graphene/gold mechanical resonator can be modeled as a capacitor with one movable plate coupled to an external voltage source. In addition to the conventional electromagnetic field energy between the capacitor plates, the system exhibits a contribution to its total energy deriving from the finite density of states (DOS) of graphene. The dependence of the graphene energy level structure on the external magnetic field, allows us to infer the magnetic properties (the susceptibility, in particular) from the measurement of the mechanical resonances as a function of *B*. The first consequence of the finite DOS of graphene is a reduction of the force between the plates of the movable capacitor *F* ≐ ∂Ω/∂*z* = 1/2*C*_g_^′^ (*V*_g_ – μ/*e*)^2^, where *V*_g_ is the external applied voltage, μ the graphene chemical potential, *C*_g_ the (position-dependent) geometric capacitance of the structure, and *C*_g_^′^ its derivative with respect to the displacement of the graphene/gold electrode (see the Supporting Information). This formula well fits the measured gate voltage dependence of the lower Au beam resonance presented in Figure 2b.

The properties of the whole system (moveable capacitor + graphene disk) can be derived from the relevant thermodynamic potential. In our case, given the *V*_g_ = constant constraint, we consider the grand canonical potential Ω(*eV*_g_, *B*, *z*), where the electrochemical potential *eV*_g_ is, the global control parameter. If we now confine ourselves to the analysis of the graphene sheet, i.e., we exclude the field between resonator and backgate from the definition of the system, we can assume that either the particle number *n* or the chemical potential μ are the control parameters. We can write the thermodynamic potential associated with the Corbino disk as Ω_disk_(*x*, *B*) = Ω_0_(*x*) + Ω_osc_(*x*, *B*), with *x* = *n*, μ. The oscillatory dependence of the thermodynamic potential Ω_osc_(*x*, *B*) on *B* is a direct consequence of the appearance of Landau levels in the energy spectrum.^2,37,38^ Central to our analysis, it is possible to write the oscillating part of the magnetic susceptibility
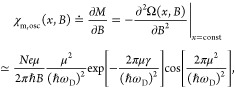
1where the second line of eq 1 corresponds to the limit  of the full expression given in eq S31b. χ_m,osc_ exhibits the oscillations characteristic of the dHvA effect, where *N* is Landau level degeneracy factor, , and γ = ℏ/(2τ_q_), with τ_q_ being the quantum scattering time (see the Supporting Information and ref (37)). As discussed in the Supporting Information eq S1, the expression for χ_m,osc_ is derived assuming a (*B*-independent) Lorentzian line width for the Landau levels. The assumption for the Landau levels structure is consistent with previous theoretical work (see for example refs (37 and 39)) and provides a good fit to our experimental data. μ should be interpreted as the independent control parameter for *x* = μ. For *x* = *n*, we should interpret μ = μ(*n*) (see the Supporting Information). The connection between the full description given by Ω(*eV*_g_, *B*) and Ω_disk_(*x*, *B*) can be understood as though the external control parameter *V*_g_ determines, along with *B*, the control parameter of the graphene disk. Since *eV*_g_ = *e^2^n*(*B*)/*C*_g_ + μ, the value of the geometric capacitance interpolates between a situation in which *V*_g_ imposes the charge on the graphene disk (*C*_g_ → 0) and the case for which the external voltage fixes the chemical potential μ (*C*_g_ → ∞). For intermediate values of *C*_g_, we should interpret the chemical potential μ appearing in eq 1 as μ = μ(*eV*_g_). Since, for our devices, we have that *eV*_g_ ≫ μ, we are essentially, from the perspective of the graphene sheet, in a charge-controlled setting (Ω_osc_(*x*, *B*) = Ω_osc_(*n*, *B*)).

Through a standard thermodynamic analysis, taking into account the charging, magnetic, and elastic energy for our devices (see the Supporting Information), it is possible to show that resonant frequencies of our structures *f*_*n*_ exhibit a nontrivial dependence on *B* reflecting the emergence of Landau levels in the spectrum of graphene (Figure 4). This frequency shift can be expressed as
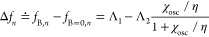
2where Λ_1_ = Λ_1_(*f*_*B*=0,*n*_,*C*_g_, *V*_g_), Λ_2_ = Λ_2_(*f*_*B*=0,*n*_,*C*_g_, *V*_g_), and η = η(*f*_*B*=0,*n*_) are given in the Supporting Information. The explicit expression of Λ_1_ and Λ_2_ allows us to establish the optimal value of the gate voltage  leading to the maximum frequency shift. While we have expressed here the frequency shift as a function of *B* in terms of χ_osc_, we can, alternatively, express it in terms of quantum capacitance (as done, for instance, in ref.^18^). The relation between the two interpretations is rooted in the (universal) thermodynamic relation that holds between quantum capacitance and magnetic susceptibility, which can be expressed as  (see the Supporting Information), allowing us to access experimentally both magnetic and charge properties of the system under consideration.

**Figure 4:**
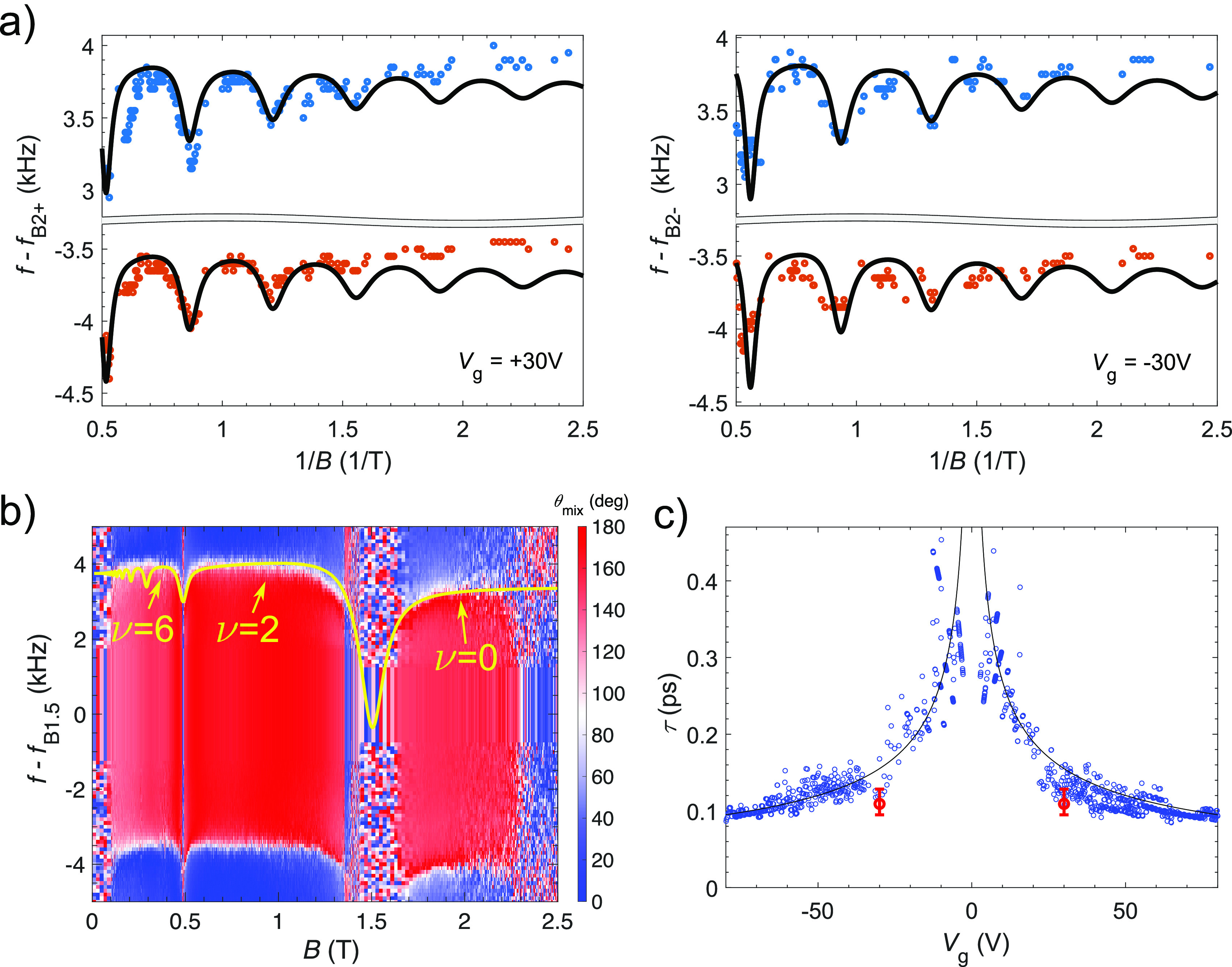
Mechanical resonance frequency shift due to de Haas–van Alphen effect. (a) Upper (blue dots) and lower (red dots) edges of the 35 MHz resonance in the B2 device at gate voltages *V*_g_ = ± 30 V as a function of 1/*B* (*f*_B2+_ = 35.28495 MHz, *f*_B2–_ = 35.28205 MHz). The edge points correspond to the frequencies where the phase of the mechanically induced mixing current flips by 180 deg (see inlay panel of Figure 3). The solid lines denote the theoretical fits with scattering times of τ_q_ ≈ 0.11 ps at *V*_g_ = ± 30 V. (b) Mixing current phase (θ) of the 26.5 MHz resonance in the device B1.5 presented as a function of *B* (*f*_B1.5_ = 26.49225 MHz). The yellow line depicts the theoretical estimate with τ_q_ ≈ 0.19 ps scattering time. (c) Quantum scattering time τ_q_ extracted from dHvA measurement in Figure 4a (red markers), and the equivalent time τ_S_ from SdH oscillations in Figure 2a (blue markers). The red error bars show a 15% deviation from the chosen Landau level widths γ in Figure 4a that still reproduces a good agreement between the theory and the experiment. The solid black line denotes  trend. The scattering time dependence on *V*_g_ implies that the LL levels become harder to resolve for larger values of *V*_g_ (see Figure 2a).

The universal interdependence of *C*_q_ and χ_m_ (and, consequently, of μ and *M*) is at the heart of the determination of the Landau level gap in torque magnetometry experiments.^40^

It is worth noting that the theory predictions shown in Figure 4 are explicitly derived using the energy spectrum of massless Dirac electrons  implying a Berry phase γ = ±π.^38^ As anticipated, even though gold resonances are heavily utilized, we are probing the magnetization properties of the graphene part of the structure: choosing the spectrum and Berry phase of 2D electron gas would result in a different spacing of the frequency dips. In analogy to the GMR measurements presented in ref (11), our analysis bears the signature of the π Berry phase characteristic of graphene.

In our measurements, we observed frequency shifts Δ*f* in the graphene/Au resonators, corresponding to the transition between QH states, consistently with eq 2. Figure 4a displays the magnetic field dependence of the lobe edges (i.e., the dips in the frequency response depicted in the inlay panel of Figure 3, corresponding to the frequencies at which phase flips for the mixing current occur). The frequency separation of these dips is related to the line width of the resonance; the data were obtained in device B2 at *V*_g_ = ± 30 V. The overlaid traces are calculated according to the theoretical model for the dHvA effect given by eq 2. The data indicate equivalent dHvA behavior for electrons and holes, which was also verified at other gate voltage values. The extracted quantum scattering time  reduces as *V*_g_ increases, which is corroborated by a similar behavior in our other devices. We resolve Δ*f* down to ∼25 Hz, but for some magnetic field ranges, e.g., around ν = 2 in Figure 4b, the magnitude of the frequency shift is not observable due to the low conductivity at the incompressible QH states. Another low-conductivity regime in Figure 4b is seen above 2.3 T, related to the state ν = 0. In addition to the integer QH states, several fractional QH states are observed in these devices,^31^ bearing witness to the sensitivity of our detection scheme.

In Figure 4b, we present the phase of the mixing current measured in the device B1.5 at the 26.5 MHz resonance at *V*_g_ = 7 V as a function of the perpendicular magnetic field. The overlaid curve is calculated from eq 2, in which two fitting parameters were employed: the voltage-dependence of the mechanical resonant frequency ∂*f*_0,*n*_/∂V = 35 kHz/V and the quantum scattering time τ_q_ = 0.19 ps. Equally good agreement is obtained for the lower phase flip as the width of the middle region (line width of the resonance) is unchanged across the measured magnetic field range. The fitting value of the scattering time is close to τ_S_ = 0.3 ps extracted previously from the Shubnikov–de Haas oscillations at *V*_g_ = 10 V in a similar device,^30^ and also close to the data in Figure 4c.

In Figure 4c, the scattering times τ_q_ obtained from the dHvA fits are plotted as a function of *V*_g_ along with the values τ_*S*_ obtained from the Shubnikov–de Haas oscillations, present in the Landau fan plot in Figure 2a. At small charge density, our value for τ_S_ matches with the scattering time obtained in ref (41) for ultraclean suspended graphene. The correspondence between the experimentally determined values of τ_q_ and τ_S_ corroborates the interpretation that our measured frequency shifts in *f*(*B*, *V*_g_) indeed arise from the dHvA effect in graphene.

Compared to the other mechanical resonance measurements with graphene samples in magnetic fields,^18,19^ our approach is different as we probe the graphene via a Au beam resonator. In a way our work is similar to the cantilever experiments by Harris and co-workers;^15^ however, by using an upper gold beam, enabling the contact with the inner edge of the Corbino geometry, we can facilitate operation on any conducting 2D material and obtain extraordinary sample quality via current annealing. This achievement seems out of reach for regular cantilever devices combined with the present state-of-the-art nanofabrication possibilities for 2D material. From this perspective, our setup opens up new possibilities in relation to the investigation of the magnetic properties of transition metal dichalcogenides (see for example refs (42−44)), with particular reference to the role of local symmetry breaking in the appearance of magnetic moments and hidden interactions in centrosymmetric crystals.^45−47^

The specific advantage of our setup consists of the fact that the relevant mechanical resonances are the gold mechanical resonances, with the “sample”, in this case graphene, mechanical resonances not playing any significant role. This offers specific advantages over a “sample-only” mechanical resonator: (1) Reproducibility of the mechanical resonant frequency is independent of the material considered, avoiding potential detrimental effects related to impurities on the resonant properties of the structure. In fact, the graphene resonance in Corbino geometry did split in several local resonances, which made studies using ”pure” graphene oscillation very challenging. (2) Larger ”dynamical range” for *V*_FM_; the layer thickness of the Au structure allows for a larger drive voltage range for which the linear mechanical regime is valid.

In conclusion, we have developed a versatile system of coupled resonators in which a Au resonator can be employed for sensing of forces originating in atomically thin suspended samples, made of graphene in the QH regime in our case. Owing to the free suspension of our graphene membrane, movement of the Au sensing element can be detected via displacement of the graphene, which facilitates force sensitivity sufficient to observe magnetization oscillations due to the de Haas–van Alphen effect in integer QH states, and even in the fractional QH regime. The experimental approach developed in this work opens up the possibility to investigate de Haas–van Alphen effect in other 2D materials, in particular transition metal dichalcogenide crystals with hidden magnetic properties.

## Supporting Information Available

Details pertaining to the theoretical analysis, the sample fabrication, and measurements (PDF)
FEM simulations of the mechanical modes (ZIP)
