# Supplementary material for: Mechanical Detection of the De Haas–van Alphen Effect in Graphene
Source: Nano Lett. 2022 Dec 13;22(24):9869–75. doi: 10.1021/acs.nanolett.2c02655 (PMC9801430; doi:10.1021/acs.nanolett.2c02655)
Supplement: Supplementary file 2 — nl2c02655_si_002.pdf [file nl2c02655_si_002.pdf]

Mechanical detection of the De Haas–van Alphen effect in graphene – Supporting information

Juuso Manninen, Antti Laitinen, Francesco Massel, and Pertti Hakonen*

E-mail: pertti.hakonen@aalto.fi

Thermodynamic analysis and forces acting on a movable capacitor (finite DOS)

Let us consider the energy per unit area of a capacitor with one of the plates constituted by a finite-DOS material (in our case graphene), in the presence of an external magnetic field B

$$U\left(n,B\right)=\frac{1}{2}\frac{e^2n^2}{C_{\mathrm{g}}}+\Xi\left(n,B\right). \tag{S1}$$

The first term in Eq. (S1) is the energy associated with the electrical field building up between the plates of the capacitor, whereas the second corresponds to the energy related to the finite density of states (DOS) of the system. In this description, the charge n on the capacitor plates and the magnetic field B are the control parameters.

Considering a standard thermodynamic relation,[1] we can define the electrochemical potential as

$$\bar{\mu}=\left(\frac{\partial U}{\partial n}\right)_{z,B}=\frac{e^2n}{C_{\mathrm{g}}}+\mu\left(n\right) \tag{S2}$$

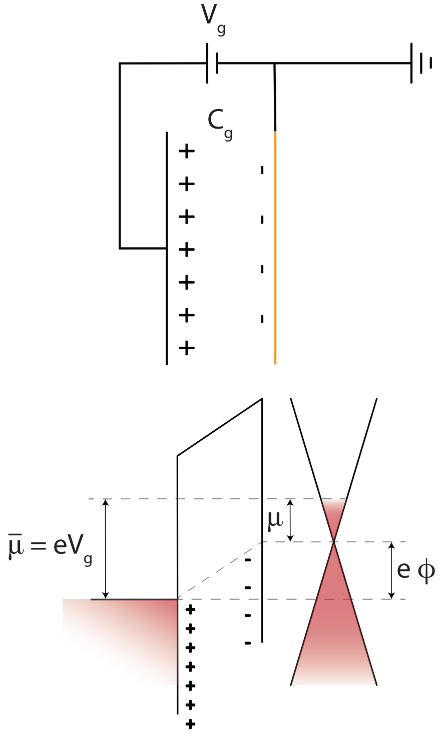

Figure S1: Top. Lumped-elements description of a capacitor with one plate constituted by a finite-DOS material (yellow) to which a voltage V_g is applied. Bottom. The voltage drop leads to the buildup of an electric field between the capacitor plates, corresponding to an electrostatic potential drop ϕ. Owing to the finite DOS, part of the energy provided by the voltage source goes into promoting electrons to higher-energy single-particle states (increase of the chemical potential μ).

implying $n = \frac{C_{\rm g}}{e^2}(\bar{\mu} - \mu)$. This relation, written as

$$\frac{\bar{\mu}}{e} = \frac{en}{C_{\rm g}} + \frac{\mu}{e},$$

allows us to show that, for $C_{\rm g} \to \infty$, the tunability of the electrochemical potential $\bar{\mu}$ is directly translated into the tunability of the chemical potential of the membrane μ. Here $\bar{\mu}$ is the electrochemical potential consisting of the electrostatic $e\phi = \frac{e^2 n}{C_{\rm g}}$ and chemical $\mu = \frac{\partial \Xi}{\partial n}$ potentials.[2]

We can now consider the thermodynamic potential $\Omega(\bar{\mu})$ where $\bar{\mu} = eV_{\rm g}$ is the independent control parameter determined by an external voltage source $V_{\rm g}$, along with B. From equation (S1), we obtain

$$\Omega(\bar{\mu}, B) = U(n, B) - \bar{\mu} n = \frac{1}{2}\frac{C_{\rm g}}{e^2}(\bar{\mu} - \mu)^2 + \Phi(\bar{\mu}, z) - \bar{\mu} n, \tag{S3}$$

where we have defined $\Phi(\bar{\mu}, B) \doteq \Xi(n(\bar{\mu}, B))$. Using equation (S3), we can calculate the force for constant $\bar{\mu}$, i.e. at constant external applied voltage

$$V_{\rm g} \doteq \frac{\bar{\mu}}{e} = \frac{en}{C_{\rm g}} + \frac{\mu}{e}. \tag{S4}$$

To this end, let us obtain a preliminary result

$$\begin{aligned} \frac{\partial n}{\partial z} &= \frac{C'_{\rm g}}{e}\left(V_{\rm g} - \frac{\mu}{e}\right) - \frac{C_{\rm g}}{e^2}\frac{\partial \mu}{\partial n}\frac{\partial n}{\partial z} = \frac{C'_{\rm g}}{e}\left(V_{\rm g} - \frac{\mu}{e}\right) - \frac{C_{\rm g}}{C_{\rm q}}\frac{\partial n}{\partial z} \\ \Longrightarrow \frac{\partial n}{\partial z} &= \left(1 + \frac{C_{\rm g}}{C_{\rm q}}\right)^{-1}\frac{C'_{\rm g}}{e}\left(V_{\rm g} - \frac{\mu}{e}\right), \end{aligned} \tag{S5}$$

where $C'_{\rm g} = \partial C_{\rm g}/\partial z$. Equation (S5) implies, in addition, that

$$\frac{\partial \mu}{\partial z} = \frac{C'_{\rm g}}{(C_{\rm q} + C_{\rm g})}(eV_{\rm g} - \mu) \tag{S6}$$

and that the electrostatic force per unit area can be expressed as

$$F = -\frac{\partial \Omega}{\partial z} = -\frac{1}{2}C_{\rm g}'\left(V_{\rm g}-\frac{\mu}{e}\right)^2 + \frac{C_{\rm g}}{e}\left(V_{\rm g}-\frac{\mu}{e}\right)\frac{\partial \mu}{\partial n}\frac{\partial n}{\partial z} - \mu\frac{\partial n}{\partial z} + eV_{\rm g}\frac{\partial n}{\partial z} \tag{S7}$$

$$= -\frac{1}{2}C_{\rm g}'\left(V_{\rm g}-\frac{\mu}{e}\right)^2 + \frac{C_{\rm g}}{C_{\rm q}}e\left(V_g-\frac{\mu}{e}\right)\frac{\partial n}{\partial z} + e\left(V_{\rm g}-\frac{\mu}{e}\right)\frac{\partial n}{\partial z} \tag{S8}$$

$$= -\frac{1}{2}C_{\rm g}'\left(V_{\rm g}-\frac{\mu}{e}\right)^2\left(1-2\frac{1+\frac{C_{\rm g}}{C_{\rm q}}}{1+\frac{C_{\rm g}}{C_{\rm q}}}\right) \tag{S9}$$

$$= \frac{1}{2}C_{\rm g}'\left(V_{\rm g}-\frac{\mu}{e}\right)^2, \tag{S10}$$

which is consistent with the expression given in the literature about carbon nanotubes.[3,4]

As discussed in the main text, the distinguishing factor between the full thermodynamic potential $\Omega\left(\bar{\mu}=eV_{\rm g}, B\right)$ and that of the graphene disk $\Omega_{\rm disk}\left({\rm x}, B\right)$ is the control parameter $\bar{\mu}eV_{\rm g}$ versus μ or n. In Fig. S2, we have compared the behavior of the oscillatory components of magnetization $M_{\rm osc}$ and magnetic susceptibility $\chi_{\rm m,osc}$, see the derivations below, as a function of B^{-1} with $\bar{\mu}$ and μ as a control parameters.

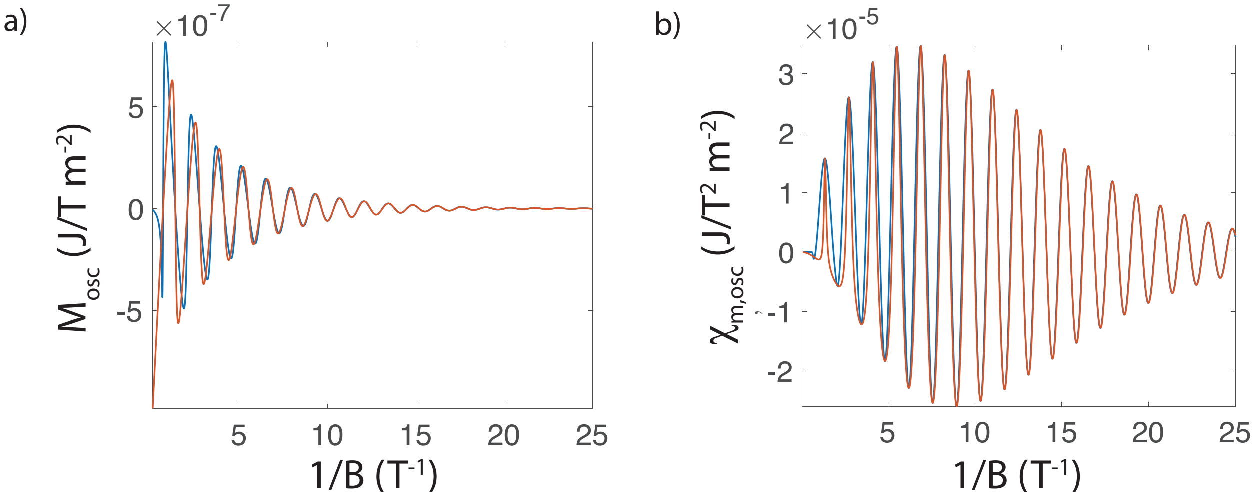

Figure S2: a) Magnetization oscillations obtained from equation S3 using $v_{\rm F}=10^6$ m/s and $\tau_q=0.19$ ps at fixed $\mu=31$ meV (red) and $\mu(B)$ for $C_{\rm g}\approx 1.15\times10^{-5}$ F/m^2, $V_{\rm g}=9.8V$ (blue). The latter case, for these parameters, essentially corresponds to a charge-controlled situation, with $n\approx 7\times10^{-14}{\rm m}^{-2}$. Lorentzian broadening with the same disorder has been considered for both plots. b) The oscillating part $\chi_{\rm m,osc}$ of magnetic susceptibility $\chi_m(\mu,B)$ for the same parameters as in panel a).

Frequency Shift

Given the expression for the force of Eq. (S10) (Eq. (1) of the main text), we evaluate here the mechanical frequency shift for a mechanical resonator whose electrical properties can be described by Eq. (S1).

For sake of simplicity, we focus here on the analysis of a membrane (vanishing flexural rigidity) in the presence of uniform tension. The only difference with the general case (finite flexural rigidity and nonuniform tension) is the specific value of the mechanical frequency in absence of external applied voltage $\omega_{0,\mathrm{n}}$, which is not central to our argument.

Firstly, we notice that the distance between the plates of the capacitor –and, consequently, the capacitance C_{g}– is modified by the application of the external electrostatic potential V_{g} (see Fig. S1). The equilibrium position of the movable capacitance $\xi_{\mathrm{e}} = \xi_{\mathrm{e}}\left(V_{\mathrm{g}}, B\right)$ can be obtained from the solution of the general elastic equation for the structure (see e.g.[5] for the case of a graphene-only membrane).

The shifts of the mechanical resonant frequency $\omega_{0,\mathrm{n}}$ for $V_{\mathrm{g}} \neq 0$ can be determined considering the membrane position fluctuations around ξ_{e}. For a membrane, these fluctuations obey the following equation

$$\rho\ddot{\zeta} - T\Delta\zeta = \delta f(\mathrm{r}, t), \tag{S11}$$

where we have defined $\delta f = [f(\xi_{\mathrm{e}} + \zeta) - f(\xi_{\mathrm{e}})]$ and $T = T(\xi_{\mathrm{e}})$. The functions $\zeta(\mathrm{r}, t)$ and $\delta f(\mathrm{r}, t)$ in Eq. (S11) can be expanded as

$$\zeta(\mathrm{r}, t) = \sum_{\mathbf{n}} \zeta_{\mathrm{n}} \exp\left[-i\omega_{\mathrm{n}} t\right] A_{\mathrm{n}}(\mathrm{r}),$$
$$\delta F(\mathrm{r}, t) = \sum_{\mathbf{n}} \delta F_{\mathrm{n}} \exp\left[-i\omega_{\mathrm{n}} t\right] A_{\mathrm{n}}(\mathrm{r}),$$

where $A(\mathrm{r})$ are the normal modes associated with the boundary value problem considered.

Furthermore, approximating

$$\delta F_{\rm n} \simeq \left.\frac{\partial F}{\partial \zeta}\right|_{\xi_{\rm e}} \zeta_{\rm n},$$

we obtain

$$\rho\omega_{\rm n}^2\zeta_{\rm n} - T\lambda_{\rm n}^2\zeta_{\rm n} = \left.\frac{\partial F}{\partial \zeta}\right|_{\xi_{\rm e}} \zeta_{\rm n}, \tag{S12}$$

leading to the following expression for the frequency of a given mode n

$$\omega_{\rm n} = \sqrt{\omega_{0,{\rm n}}^2 - \frac{1}{\rho}\left.\frac{\partial F}{\partial \zeta}\right|_{\xi_{\rm e}}} \tag{S13}$$

with $\omega_{0,{\rm n}} = \sqrt{T\lambda_{\rm n}^2/\rho}$, where $\lambda_{\rm n}$ depends on the geometry considered. For the case of a disk of radius R, we have that $\lambda_{\rm n} \to \lambda_{i,j} = \alpha_{i,j}/R$, where $\alpha_{i,j}$ are the roots of the Bessel function $J_{i,j}(r)$. For the case of metallic leads (infinite DOS), we have that $\partial F/\partial\zeta) = 1/2C_{\rm g}''V_{\rm g}^2$ allowing us to obtain from Eq. (S13) the usual capacitive softening voltage dependence of the mechanical resonant frequency.

In the case of finite DOS, from Eq. (S10), we can write

$$\left.\frac{\partial F}{\partial \zeta}\right|_{\xi_{\rm e}} = \frac{1}{2}C_{\rm g}''\left(V_{\rm g} - \frac{\mu}{e}\right)^2 - C_{\rm g}'\left(V_{\rm g} - \frac{\mu}{e}\right)\frac{1}{e}\left.\frac{\partial \mu}{\partial \zeta}\right|_{\xi_{\rm e}}, \tag{S14}$$

which with the help of Eq. (S6), gives

$$\left.\frac{\partial F}{\partial \zeta}\right|_{\xi_{\rm e}} = \left\{\frac{1}{2}C_{\rm g}'' - \frac{{C_{\rm g}'}^2}{C_{\rm q} + C_{\rm g}}\right\}\left.\left(V_{\rm g} - \frac{\mu}{e}\right)^2\right|_{\xi_{\rm e}}. \tag{S15}$$

Denoting by $\Delta\omega_{\rm n}$ the difference between the resonant frequency at finite B with respect to the frequency at $B = 0$, we can write

$$\Delta\omega_{\rm n} \simeq \Delta\omega_{0,{\rm n}} - \frac{1}{2\rho\omega_{0,{\rm n}}}\left(\left.\frac{\partial F}{\partial \zeta}\right|_{B} - \left.\frac{\partial F}{\partial \zeta}\right|_{B=0}\right), \tag{S16}$$

where $\Delta\omega_{0,\mathrm{n}} = \omega_{0,\mathrm{n}}(B) - \omega_{0,\mathrm{n}}(B=0)$. When writing $\partial F/\partial\zeta|_B$, we have considered both the the explicit $B-$dependence of the derivative at $\xi_\mathrm{e} = \xi_\mathrm{e}(0)$, and the dependence of ξ_e on the magnetic field. The first term in Eq. (S16) represents the change of the resonant frequency with the external magnetic field associated with tensioning effects, whereas the second term is related to the magnetic field change of the capacitive softening term.

Noting that, for $B = 0$, the resonant frequency $\omega_{0,\mathrm{n}}$ depends on the externally applied voltage only, we can approximate

$$\Delta\omega_{0,\mathrm{n}} = \frac{\partial\omega_{0,\mathrm{n}}}{\partial V}\frac{\partial V}{\partial F}\bigg|_{B=0}\Delta F \simeq -\frac{\partial\omega_{0,\mathrm{n}}}{\partial V}\bigg|_{B=0}\frac{\Delta\mu(B,\xi_\mathrm{e})}{e}. \tag{S17}$$

Integrating equation (S6), in the limit $V_\mathrm{g} \gg \mu/e$ and $C_\mathrm{q} \gg C_\mathrm{g}$, we have

$$\mu(B,\xi) = \mu(B) + \frac{e\,C_\mathrm{g}(\xi_\mathrm{e})V_\mathrm{g}}{C_\mathrm{q}(B)}. \tag{S18}$$

The two terms in Eq. (S18) take into account the explicit dependence of μ on the external magnetic field and its dependence on ξ_e, respectively.

Equations (S17, S18) lead to

$$\Delta\omega_{0,\mathrm{n}} = -\frac{\partial\omega_{0,\mathrm{n}}}{\partial V}\left[\Delta\mu + C_\mathrm{g}V_\mathrm{g}\Delta\left(\frac{1}{C_\mathrm{q}}\right)\right]. \tag{S19}$$

Analogously, we have that

$$\frac{\partial F}{\partial\zeta}\bigg|_B - \frac{\partial F}{\partial\zeta}\bigg|_{B=0} \simeq -C''{}_\mathrm{g}V_\mathrm{g}\left[\frac{\Delta\mu}{e} + C_\mathrm{g}V_\mathrm{g}\Delta\left(\frac{1}{C_\mathrm{q}}\right)\right] - \left(C'{}_\mathrm{g}V_\mathrm{g}\right)^2\Delta\left(\frac{1}{C_\mathrm{q}}\right), \tag{S20}$$

leading to

$$\Delta\omega_\mathrm{n} = \left[-\frac{\partial\omega_{0,\mathrm{n}}}{\partial V} + \frac{C''V_g}{2\rho\omega_{0,\mathrm{n}}}\right]\left[\frac{\Delta\mu}{e} + C_\mathrm{g}V_\mathrm{g}\Delta\frac{1}{C_\mathrm{q}}\right] + \frac{\left(C'_\mathrm{g}V_\mathrm{g}\right)^2}{2\rho\omega_{0,\mathrm{n}}}\Delta\left(\frac{1}{C_\mathrm{q}}\right), \tag{S21}$$

which with $\kappa_\mathrm{n} = \rho\omega_\mathrm{n}^2$ and $\omega_\mathrm{n} = 2\pi f_\mathrm{n}$ leads to Eq. (2) of the main text. We note also that,

in the limit $C''_{\rm g} = 0$, Eq. (S21) corresponds to the expression given in[6] for the mechanical frequency shift.

Quantum capacitance

We derive here the expression for the quantum capacitance $C_{\rm q}$. To this end, we express the density of states as a sum of Lorentzians centered at $\epsilon_{\rm n} = {\rm sign}(n)\hbar\omega_{\rm D}\sqrt{|n|}$ ($\omega_{\rm D} = v_{\rm f}\sqrt{2eB/\hbar}$)

$$D(\epsilon) = \frac{NeB}{2\pi\hbar}\sum_{n=-\infty}^{\infty}\frac{\gamma}{2\pi}\frac{1}{(\epsilon-\epsilon_n)^2+\frac{\gamma^2}{4}} \tag{S22}$$

with the Landau level degeneracy factor $N = 4$. Our analysis is based on the possibility of turning an infinite sum into an integral over the complex plane. If $f(w)$ is a meromorphic function, the following condition is fulfilled

$$\sum f(n) = \oint_{\mathcal{C}_{\rm n}} \pi\cot(\pi w) f(w)dw - \sum_k {\rm Res}\left[\pi\cot(\pi w)f(w); w_k\right]. \tag{S23}$$

Since, in our case, we have

$$f(n) = \frac{NeB}{2\pi\hbar}\frac{\gamma}{2\pi}\frac{1}{\left(\omega - {\rm sign}(n)\omega_{\rm D}\sqrt{|n|}\right)^2+\frac{\gamma^2}{4}} \tag{S24}$$

we operate a change of variables $w \to z^2$, which allows us to rewrite Eq. (S23) as

$$\sum f(n) = \oint_{\mathcal{C}_{\rm n}} 2\pi z\cot(\pi z) f(z^2)dz - \sum_k {\rm Res}\left[2\pi z\cot(\pi z)f\left(z^2\right); z_k^2\right], \tag{S25}$$

see Fig. S3 for the complex-plane representation of contours and poles leading to Eq. (S25).

The integral on the lhs of Eq. (S25) represents a (diverging) constant contribution, which can be renormalized by introducing an explicit frequency cutoff, analogously to what is done

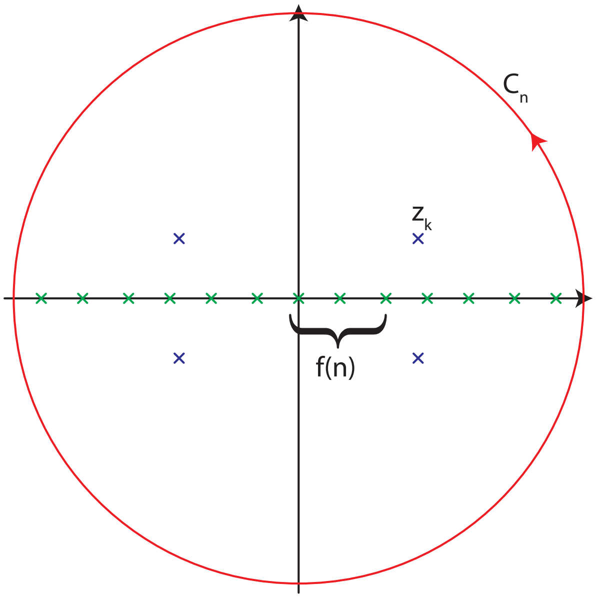

Figure S3: Complex-plane representation of the relation in Eq. (S25). The red line represents the contour along which the integral on the lhs is performed. We have denoted the poles of $2\pi z\cot(\pi z)$ (whose residue is $f(n)$) in blue, whereas the poles of $f\left(z^2\right)$ are marked in red.

in Ref.[7] Conversely, the second term, the sum over the poles of $2\pi z\cot(\pi z)f(z)$, can be evaluated explicitly, leading to the following expression for the density of states

$$D(\epsilon)=\Lambda_D+\frac{NeB}{\pi\left(\hbar\omega_{\rm D}\right)^2}\frac{\epsilon\sinh\left[\frac{2\pi\gamma\epsilon}{(\hbar\omega_{\rm D})^2}\right]-\frac{\gamma}{2}\sin\left[\frac{2\pi\left(\epsilon^2-\frac{\gamma^2}{4}\right)}{(\hbar\omega_{\rm D})^2}\right]}{\cosh\left[\frac{2\pi\gamma\epsilon}{(\hbar\omega_{\rm D})^2}\right]-\cos\left[\frac{2\pi\left(\epsilon^2-\frac{\gamma^2}{4}\right)}{(\hbar\omega_{\rm D})^2}\right]},\tag{S26}$$

where $\Lambda_{\rm D}$ is the contribution coming from the integral on the contour $\mathcal{C}_{\rm n}$.

In the $\omega\gg\gamma$, $\omega_{\rm D}$ limit the expression for $D(\omega)$ given in Eq. (S26), coincides with the one given in Ref.,[7] in the same limit. From Eq. (S26) we can easily derive the expression for $C_{\rm q}=e^2D(\mu)$.

Quantum capacitance and de Haas – van Alphen effect

With an analogous calculation to the one leading to the expression for $C_{\rm q}$, it is possible to derive an expression for the charge density n

$$n = n_0 + n_{\rm osc} \tag{S27}$$

with

$$n_0 = \frac{NeB}{2\pi\hbar}\left(\mu^2 - \frac{\gamma^2}{4}\right)/\omega_{\rm D}^2 \tag{S28a}$$

$$n_{\rm osc} = \frac{NeB}{2\pi^2\hbar}\arctan\left\{\frac{\sin\left[\frac{2\pi}{(\hbar\omega_{\rm D})^2}\left(\mu^2 - \frac{\gamma^2}{4}\right)\right]}{\exp\left[\frac{2\pi\gamma\mu}{(\hbar\omega_{\rm D})^2}\right] - \cos\left[\frac{2\pi}{(\hbar\omega_{\rm D})^2}\left(\mu^2 - \frac{\gamma^2}{4}\right)\right]}\right\} \tag{S28b}$$

and to the oscillatory component of the magnetization $M_{\rm osc} = -\frac{\partial \Omega_{\rm osc}}{\partial B}$

$$M_{\rm osc} = -\frac{Ne}{4\pi^2\hbar}\mu\arctan\left\{\frac{\sin\left[\frac{2\pi}{(\hbar\omega_{\rm D})^2}\left(\mu^2 - \frac{\gamma^2}{4}\right)\right]}{\exp\left[\frac{2\pi\gamma\mu}{(\hbar\omega_{\rm D})^2}\right] - \cos\left[\frac{2\pi}{(\hbar\omega_{\rm D})^2}\left(\mu^2 - \frac{\gamma^2}{4}\right)\right]}\right\}. \tag{S29}$$

The relations given by Eqs. (S27,S28aS28b, complemented by the relation

$$\bar{\mu} = eV_{\rm g} = \frac{e^2 n}{C_{\rm g}} + \mu \tag{S30}$$

allow us to determine (numerically, for finite values of $C_{\rm g}$) the dependence of μ and n on $\bar{\mu} = eV_{\rm g}$. For $C_{\rm g} \to 0$ and $C_{\rm g} \to \infty$, as mentioned in the main text, it is possible to consider the charge n or the chemical potential μ as control parameters for the graphene disk (see also[7]). From Eqs. (S28aS28b, S29) is straightforward to demonstrate a relation between the oscillating component of the quantum capacitance $C_{\rm q,osc} = e^2\partial n_{\rm osc}/\partial\mu$ and the oscillations

of the magnetic susceptibility $\chi_{\mathrm{m,osc}} = \frac{\partial M_{\mathrm{osc}}}{\partial B}$. From Eqs. (S28a-S29), we have that

$$C_{\mathrm{q,osc}} = -e^2 \frac{NeB}{\pi\hbar} \frac{\mu}{(\hbar\omega_{\mathrm{D}})^2} \frac{\left\{e^{-2\pi\gamma\mu/(\hbar\omega_{\mathrm{D}})^2} - \cos\left[\frac{2\pi}{(\hbar\omega_{\mathrm{D}})^2}\left(\mu^2 - \frac{\gamma^2}{4}\right)\right]\right\} + \gamma/2\sin\left[\frac{2\pi}{(\hbar\omega_{\mathrm{D}})^2}\left(\mu^2 - \frac{\gamma^2}{4}\right)\right]}{\cosh\left[\frac{2\pi\gamma\mu}{(\hbar\omega_{\mathrm{D}})^2}\right] - \cos\left[\frac{2\pi}{(\hbar\omega_{\mathrm{D}})^2}\left(\mu^2 - \frac{\gamma^2}{4}\right)\right]}, \tag{S31a}$$

$$\chi_{\mathrm{m,osc}} = -\frac{Ne\mu}{4\pi\hbar B} \frac{\left(\mu^2 - \frac{\gamma^2}{4}\right)\left\{e^{-2\pi\gamma\mu/(\hbar\omega_{\mathrm{D}})^2} - \cos\left[\frac{2\pi}{(\hbar\omega_{\mathrm{D}})^2}\left(\mu^2 - \frac{\gamma^2}{4}\right)\right]\right\} + \gamma\mu\sin\left[\frac{2\pi}{(\hbar\omega_{\mathrm{D}})^2}\left(\mu^2 - \frac{\gamma^2}{4}\right)\right]}{(\hbar\omega_{\mathrm{D}})^2\left\{\cosh\left[\frac{2\pi\gamma\mu}{(\hbar\omega_{\mathrm{D}})^2}\right] - \cos\left[\frac{2\pi}{(\hbar\omega_{\mathrm{D}})^2}\left(\mu^2 - \frac{\gamma^2}{4}\right)\right]\right\}}, \tag{S31b}$$

which leads to the relation

$$C_{\mathrm{q,osc}} = \frac{\chi_{\mathrm{m,osc}}}{\Gamma(\mu, B)} \tag{S32}$$

with

$$\Gamma(\mu, B) = \frac{\mu^2 - \gamma^2/4}{4B^2e^2} + \frac{\gamma}{4B^2e^2} \frac{\mu^2 + \gamma^2/4}{\gamma + 2\mu\sin\left[\frac{2\pi}{(\hbar\omega_{\mathrm{D}})^2}\left(\mu^2 - \frac{\gamma^2}{4}\right)\right]^{-1}\left\{\cos\left[\frac{2\pi}{(\hbar\omega_{\mathrm{D}})^2}\left(\mu^2 - \frac{\gamma^2}{4}\right)\right] - \exp\left[\frac{2\pi\gamma\mu}{(\hbar\omega_{\mathrm{D}})^2}\right]\right\}}. \tag{S33}$$

Here for $\mu \gg \gamma$, we obtain

$$\Gamma(\mu, B) = \left(\frac{\mu}{2eB}\right)^2. \tag{S34}$$

We note here that Eq. (S32) can be obtained from a general thermodynamic argument. Considering the two pairs of intensive/extensive variables (n, μ), (M, B), we can define four possible thermodynamic potentials. We focus here on the choice $G = G(n, B)$, which allows

us to define

$$\mu = \left.\frac{\partial G}{\partial n}\right|_B, \; M = -\left.\frac{\partial G}{\partial B}\right|_n \tag{S35}$$

From the chain rule of the derivative, considering that μ and M can be intepreted as independent variables –this is in fact the choice we have operated above when considering the thermodynamics potential $\Omega = \Omega(\mu, M)$– we have

$$\left.\frac{\partial \mu}{\partial n}\right|_B \left.\frac{\partial n}{\partial M}\right|_\mu + \left.\frac{\partial \mu}{\partial B}\right|_n \left.\frac{\partial B}{\partial M}\right|_\mu = 0, \tag{S36}$$

allowing us to write

$$\frac{e^2 \chi_{\rm m}}{C_{\rm q}} = -\left.\frac{\partial \mu}{\partial B}\right|_n \left.\frac{\partial M}{\partial n}\right|_\mu \tag{S37}$$

which, considering the Maxwell relation $\left.\partial M/\partial n\right|_\mu = -\left.\partial \mu/\partial B\right|_n$, leads to

$$\frac{e^2 \chi_{\rm m}}{C_{\rm q}} = \left(\left.\frac{\partial \mu}{\partial B}\right|_n\right)^2. \tag{S38}$$

Eq. (S38) establishes a general relation between quantum capacitance and magnetic susceptibility, which, for graphene, in the limit $\mu \gg \gamma$, corresponds to the expression given in Eq. (S34).

Neglecting the small oscillations of the chemical potential, it is therefore clear that the dips in the dependence of the mechanical resonant frequency $\omega_{\rm n}$ as a function of B, which were previously been interpreted in terms of quantum capacitance oscillations, can equivalently be interpreted in terms of oscillations of the magnetic susceptibility $\chi_{\rm m}$ (de Haas – van Alphen effect).

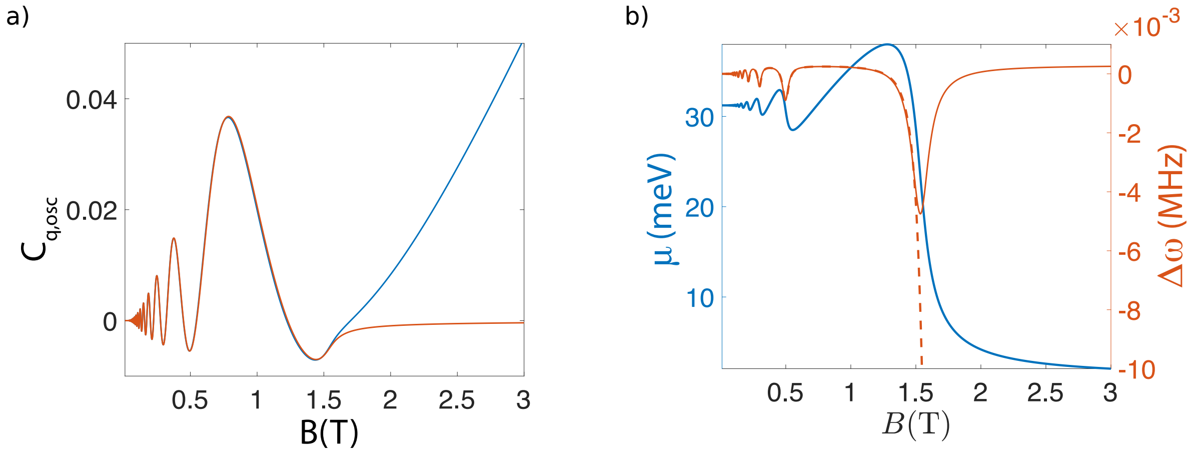

Figure S4: a) Value of $C_{\mathrm{q,\,osc}}$ calculated through the relation $C_{\mathrm{q,osc}} = \frac{\chi_{\mathrm{m,osc}}}{\Gamma(\mu,B)}$, for the value of $\Gamma(\mu, B)$ given in equation (S33) (blue line), and for the zeroth-order expansion in γ (equation (S34), red line). b) Corresponding plot of the frequency shift. Exact value of $\Gamma(\mu, B)$ (continuous line), zeroth-order expansion in γ (dotted line). All parameters are the ones used for the data fit of device B1.5.

From Eq. (S32) we can write

$$\Delta \frac{1}{C_{\mathrm{q}}} = \frac{1}{C_{\mathrm{q}}} - \frac{1}{C_{\mathrm{q0}}} = -\frac{\chi_{\mathrm{m,osc}}/\Gamma}{C_{\mathrm{q0}}\left(C_{\mathrm{q0}} + \chi_{\mathrm{m,osc}}/\Gamma\right)} \tag{S39}$$

Substituting Eq. (S39) into Eq. (S21) we obtain

$$\Delta\omega_{\mathrm{n}} = \left[-\frac{\partial\omega_{0,\mathrm{n}}}{\partial V} + \frac{C''V_g}{2\rho\omega_{0,\mathrm{n}}}\right]\frac{\Delta\mu}{e} + \left\{\left[\frac{\partial\omega_{0,\mathrm{n}}}{\partial V} - \frac{C''V_g}{2\rho\omega_{0,\mathrm{n}}}\right]C_{\mathrm{g}}V_{\mathrm{g}} - \frac{\left(C'_{\mathrm{g}}V_{\mathrm{g}}\right)^2}{2\rho\omega_{0,\mathrm{n}}}\right\}\frac{\chi_{\mathrm{m,osc}}/\Gamma}{C_{\mathrm{q0}}\left(C_{\mathrm{q0}} + \chi_{\mathrm{m,osc}}/\Gamma\right)} \tag{S40}$$

which, upon defining

$$\Lambda_1 = \left[-\frac{\partial\omega_{0,\mathrm{n}}}{\partial V} + \frac{C''V_g}{2\rho\omega_{0,\mathrm{n}}}\right]\frac{\Delta\mu}{e}$$

$$\Lambda_2 = \frac{1}{C_{\mathrm{q0}}}\left\{\left[\frac{\partial\omega_{0,\mathrm{n}}}{\partial V} - \frac{C''V_g}{2\rho\omega_{0,\mathrm{n}}}\right]C_{\mathrm{g}}V_{\mathrm{g}} - \frac{\left(C'_{\mathrm{g}}V_{\mathrm{g}}\right)^2}{2\rho\omega_{0,\mathrm{n}}}\right\}$$

$$\eta = C_{\mathrm{q0}}\Gamma,$$

leads to Eq. (2) of the main text, where $C_{q,0} = e^2 \partial n_0/\partial\mu = Ne^2\mu/(\pi v_F^2 \hbar^2)$. Close to the frequency dips, $\Lambda_1 \approx 0$ and, given the large elastic constant of gold, $\Lambda_2 \approx \frac{1}{C_{q0}}\left[\frac{\partial\omega_{0,n}}{\partial V} C_g V_g - \frac{\left(C_g' V_g\right)^2}{2\rho\omega_{0,n}}\right]$. From the value of Λ_2 it is possible to establish the optimal gate voltage for the observation of the frequency dips, which is given by

$$V_g = \frac{C_g}{C_g'} \frac{k_0}{\omega_0} \frac{\partial\omega_0}{\partial V_g} \tag{S41}$$

Sample fabrication and measurement setting

Our Au resonators with suspended graphene Corbino disks were fabricated using a method adapted from Ref.[8] and described in depth in Ref.[9] The fabrication was based on selective manipulation of the two different resists with electron beam: PMMA for defining metal contacts, and lift-off-resist (LOR) for support of suspended structures. The Cr/Au contacts were deposited using ultra-high vacuum metal evaporation in two steps with an LOR layer spun to separate the bottom contact defining the outer rim of the Corbino device, while the top contact supported by the separation layer bridged to the middle of the Corbino ring. The thickness of the Au electrodes amounted to $h_L = 70$ nm (the lower electrode) and $h_U = 120$ nm (the upper electrode).

The whole structure was supported by $t_V = 500$ nm of LOR standing on a standard $t_{Si} = 300$ nm thick SiO_2 covered Si++ substrate that served as a back gate. Thus, the vacuum gap (thickness t_V) and SiO_2 as insulating layers yield $d_{eff} = t_V + t_{Si}/\epsilon_r = 580$ nm for the effective gap of the gate capacitor. This corresponds to the geometric gate capacitance value $C_g \approx 1.15 \times 10^{-5}$ F/m^2 obtained from the measured Landau fan diagram. Furthermore, the mechanical resonant frequencies at $B = 0$ exhibit the expected capacitive softening behavior. Due to the built-in strain and a rather large distance between the graphene and the gate electrodes, our graphene membranes easily sustain voltages up to $V_g = 100$ V.

The fabricated sample chips were glued into sample boxes with microwave striplines that in turn could be wire bonded to the bonding pads of the Corbino devices. The sample boxes

were connected to the measurement lines through bias tees that allowed the DC conductance measurements and low frequency readout of the mechanical resonance, as well as the high frequency RF input used to transduce the mechanical motion.

The results presented here were obtained on two samples, B1.5 and B2, in which the Au beams were connected graphene Corbino disks with outer (inner) diameter of 3.8 (1.5) and 4.5 (1.8) μm, respectively. The length of the main, lower Au resonator, connected to the outer rim of graphene, was approximately 8 μm, with a cross section of 70 nm $\times$ 5 μm. The best results were obtained using resonance frequencies around $25 - 35$ MHz, which corresponds to the third harmonic our 8-μm-long Au beam. Quality factors of these resonances amounted to ~ 4000.

The fabricated devices were characterized using standard conductance measurement techniques and resonance measurements at 10 mK. The devices were mounted slightly off center of the 9 T magnet on a Bluefors LD400 dilution refrigerator. At the sample location, $dB/dz = 60$ T/m and $d^2B/dz^2 = 1100$ T/m^2 with a maximum field of 6.8 T. This second derivative has such a tiny effect on the Au mechanical frequencies so that it can be neglected in our force analysis.

Prior to the actual measurements, however, current annealing[10] was performed by applying a bias voltage $V_b \approx 2$ V across the Corbino ring, consequently evaporating residues from fabrication off from the graphene flake. The device quality was assessed by measuring the Landau fan diagram, such diagram is presented in Fig. 2a for the investigated sample B2. Note the fractional QH state $\nu = 1/3$ is visible from $B \approx 3$ T upwards along with the usual set of integer quantum Hall states highlighting the good quality of the measured samples. At higher fields more fractional states appeared, see Refs.[9,11].

Identification of quantum Hall states

Detection of the QH states in the graphene Corbino was performed both using low-frequency AC conductance and the mechanical response of the combined gold-graphene modes. The

sensitive Au resonance detection of QH states via graphene's mechanical response is facilitated by the variation of the derivative dG/dV_g that specifies the magnitude of the mixing current I_mix in graphene (see Eq. (S42)). Consequently, I_mix pinpoints regions with $dG/dV_\mathrm{g} = 0$, across which the mixing current changes its sign. In the experiment, the sign change of I_mix is seen as a flip of the phase by π in the down-mixed signal. Fig. 3 displays the measured conductance $G(V_\mathrm{g})$ and the phase of the mixing current. An exact match between $dG/dV_\mathrm{g} = 0$ locations and the phase flips is observed.

Mechanical resonances

Mechanical resonances were detected using the FM mixing technique.[12] In this technique an FM-modulated signal $V^\mathrm{FM}(t) = V^\mathrm{AC}\cos(2\pi f_\mathrm{c} t + (f_\Delta/f_\mathrm{L})\sin(2\pi f_\mathrm{L} t))$ was fed to one of the electrodes of the Corbino device through a bias tee. Here V^AC and f_c are the carrier amplitude and frequency, respectively. The sinusoidal low-frequency modulation signal at f_L (typically $\sim$ 600 Hz) was supplied by the SR830 lockin amplifier, while the frequency deviation f_Δ (typically 1-4 kHz) was produced by the frequency generator (Rohde & Schwarz SMY01 or Keysight N9310A for the B2 sample ,and HP8656B for the B1.5 sample) producing the FM-modulated signal.

The FM-modulated signal, applied across source-drain electrodes of the graphene membrane, got downmixed by the intrinsic nonlinearity of the graphene device, and the low frequency component at the frequency f_L reflects the mechanical motion amplitude z of the graphene flake. This proportionality can be expressed as

$$I_\mathrm{mix} \propto \frac{C'_\mathrm{tot}}{C_\mathrm{tot}} \frac{\partial G}{\partial V_\mathrm{g}} \left| \frac{\partial \mathrm{Re}(z)}{\partial f} \right|, \tag{S42}$$

where $C_\mathrm{tot} = (1/C_\mathrm{g} + 1/C_\mathrm{q})^{-1}$ is the total capacitance, and $C'_\mathrm{tot} = \frac{dC_\mathrm{tot}}{dz}$. C_g and C_q are the gate capacitance and the quantum capacitance per unit area, respectively. Phase shifts may occur between the drive and the response due interference phenomena in the flexural waves

traveling along the Corbino disk, driven from the outer edge. In the case of phase shifts, the mechanical response function $\frac{\partial \mathrm{Re}(z)}{\partial f}$ will obtain a corresponding reference phase, which results in a combination of dispersive and absorptive parts of the mechanical response. The downmixed signal from the other electrode of the corbino disk was led back to the lock-in amplifier through a Stanford SR570 current amplifier with gain 10^6 V/A.

The observed combined gold-graphene modes below $\sim$ 40 MHz involve either the lower or upper Au electrode beam, the motion of which is followed by graphene at master-slave principle owing to the time-dependent boundary conditions imposed by Au on graphene. The 35.3 MHz resonance of the B2 device depicted in Fig. 4a, for example, is detected with the measurement configuration shown in Fig. 1b but is not observable when the source and drain sides are reversed implying that this mechanical mode is dominated by the movement of the lower gold beam with the graphene sheet following. Moreover, it is this exclusive reliance on the mechanical Au resonances in actuation and detection, regardless of the properties of graphene, that allows us to generalize our investigation method to other 2D materials. Overhead views of the mode shapes arising from finite element simulations, performed with COMSOL Multiphysics, for the mechanical modes discussed in Fig. 4ab in the main text are shown in Fig. S5.

In our frequency sweeps of the sample B1.5, we observed 12 mechanical modes below 27 MHz. Using COMSOL simulations, candidate mode shapes for these modes could be identified. We utilized simulated gate voltage dependencies for each mode to determine the mode shape corresponding to the 26.5 MHz resonance with which the dHvA effect was observed. A mode shape, where the most significant role is played by the cantilever, displays a weak frequency increase with respect to the gate voltage in the simulations corresponding to the observed trend in the measurements.

We emphasize that our detection scheme for magnetization effects in a 2D material relies on finding well-defined resonances of the gold structure and is, therefore, suitable for a very wide variety 2D systems. Specifically, the 2D-material portion of the structure is not required

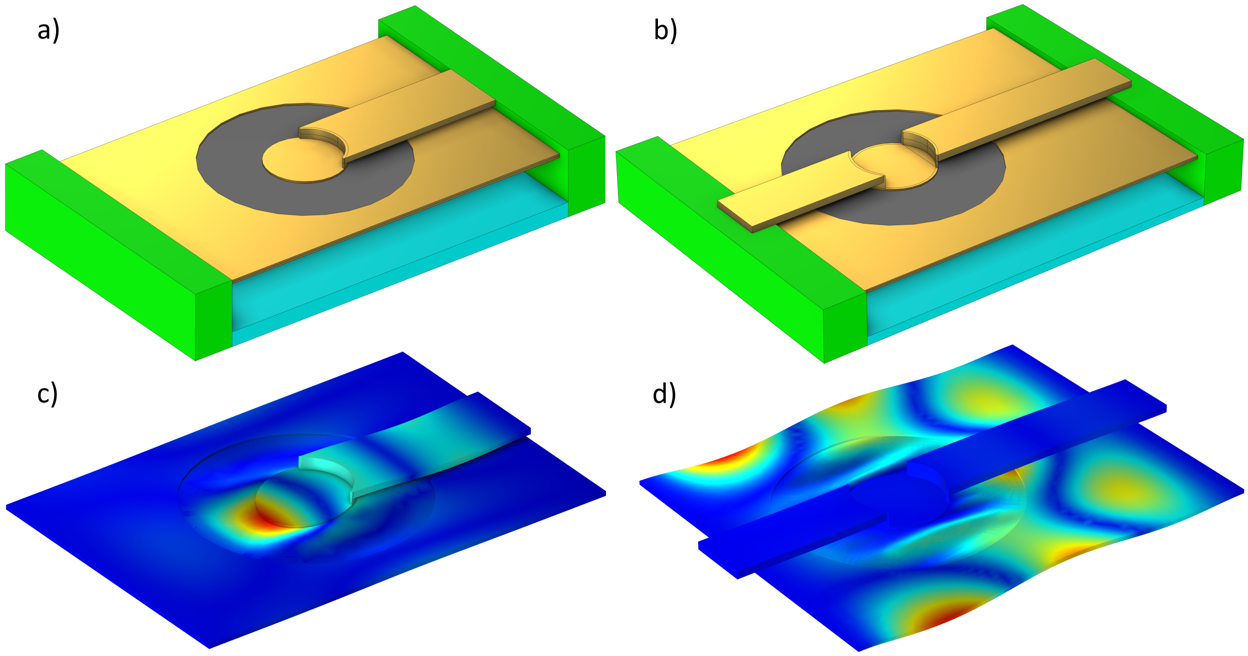

Figure S5: An overhead view of resonator structures (a,b) and mode shapes (c,d) arising from the COMSOL finite element simulations for the 26.5 MHz mode of device B1.5 (a,c) and the 35 MHz mode of device B2 (b,d).

to have good resonator properties. For example, in our case, the resonances shown here have quality factors of the order of 1000.

In addition to graphene and Au modes, surface waves around 20 MHz were excited in the LOR-layer by the microwave drive.[13] Even though these modes could be excited at very small power, they were not useful for detection purposes owing to the small $Q = 100 - 200$.

Fitting the theory predictions

The theoretical predictions of the frequency shift due to the dHvA effect given in Eq. 2 are fitted to the experimental data as shown in Figs. 4a and 4b. To obtain the spring constant of the modes, we assume that the effective mass of the modes is determined by the gold resonator, and graphene's contribution is practically negligible. We approximate that, for the B2 device $\sim 80\,\mathrm{nm}$ thick lower gold beam, the 2D density is $\rho \approx 1.5 \times 10^{-3}\,\mathrm{kg/m^2}$, and $\rho \approx 2.1 \times 10^{-3}\,\mathrm{kg/m^2}$ for the $\sim 110\,\mathrm{nm}$ thick cantilever of B1.5.

For our setup, the last term of the frequency shift in Eq. 2 is negligible and, therefore, the size of the frequency shift is scaled by the factor $\partial f_{0,\mathrm{n}}/\partial V$. We fit $\partial f_{0,\mathrm{n}}/\partial V$ to the experimental data to obtain proper magnitude of Δf together with the Landau level width γ that affects the magnitude of Δf as well as the width of the frequency dips. For the device B2 (B1.5) we have 16 kHz/V (35 kHz/V). The larger value for the B1.5 device mode is expected due to it being a mode of the cantilever whose other end is attached to the graphene. This boundary condition makes the B1.5 mode more sensitive to tensioning effects than the resonance of the lower gold plate of the B2 device.

The scattering time $\tau_S = \hbar\sqrt{\pi n}\mu_q/ev_{\mathrm{F}}$ shown in Fig. 3c was calculated from the quantum mobility μ_q, which in turn was obtained by extracting the minimum field of Shubnikov–de Haas oscillations $B_0(V_{\mathrm{g}})$ and using the relation $\mu_q B_0 = 1$. Additionally, the error bars of τ_{q} in the same figure show a 15% deviation from the values of $\gamma = \hbar/(2\tau_{\mathrm{q}})$ used to fit the theory curves in Fig. 3a. Values of γ within this tolerance reproduce a good agreement of Δf between the theory and the experiment.

References

(1) Callen, H. B. *Thermodynamics and an Introduction to Thermostatistics*; John Wiley & Sons; John Wiley & Sons, 2006; pp 153–199.

(2) Yu, G. L.; Jalil, R.; Belle, B.; Mayorov, A. S.; Blake, P.; Schedin, F.; Morozov, S. V.; Ponomarenko, L. A.; Chiappini, F.; Wiedmann, S.; Zeitler, U.; Katsnelson, M. I.; Geim, A. K.; Novoselov, K. S.; Elias, D. C. Interaction phenomena in graphene seen through quantum capacitance. *Proceedings of the National Academy of Sciences* **2013**, *110*, 3282 – 3286.

(3) Steele, G. A.; Hüttel, A. K.; Witkamp, B.; Poot, M.; Meerwaldt, H. B.; Kouwenhoven, L. P.; van der Zant, H. S. J. Strong Coupling Between Single-Electron Tunneling and Nanomechanical Motion. *Science* **2009**, *325*, 1103–1107.

(4) Lassagne, B.; Tarakanov, Y.; Kinaret, J.; Garcia-Sanchez, D.; Bachtold, A. Coupling Mechanics to Charge Transport in Carbon Nanotube Mechanical Resonators. *Science* **2009**, *325*, 1107–1110.

(5) Atalaya, J.; Isacsson, A.; Kinaret, J. M. Continuum Elastic Modeling of Graphene Resonators. *Nano Letters* **2008**, *8*, 4196–4200.

(6) Chen, C.; Deshpande, V. V.; Koshino, M.; Lee, S.; Gondarenko, A.; MacDonald, A. H.; Kim, P.; Hone, J. Modulation of mechanical resonance by chemical potential oscillation in graphene. *Nature Phys.* **2016**, *12*, 240.

(7) Sharapov, S. G.; Gusynin, V. P.; Beck, H. Magnetic oscillations in planar systems with the Dirac-like spectrum of quasiparticle excitations. *Phys. Rev. B* **2004**, *69*, 075104.

(8) Tombros, N.; Veligura, A.; Junesch, J. J., J. van den Berg; Zomer, P. J.; Wojtaszek, M.; Vera Marun, I. J.; Jonkman, H. T.; van Wees, B. J. Large yield production of high mobility freely suspended graphene electronic devices on a polydimethylglutarimide based organic polymer. *J. Appl. Phys.* **2011**, *109*, 093702.

(9) Kumar, M.; Laitinen, A.; Hakonen, P. Unconventional fractional quantum Hall states and Wigner crystallization in suspended Corbino graphene. *Nature Communications* **2018**, *9*.

(10) Moser, J.; Barreiro, A.; Bachtold, A. Current-induced cleaning of graphene. *Applied Physics Letters* **2007**, *91*, 163513.

(11) Laitinen, A.; Kumar, M.; Hakonen, P. J. Weak antilocalization of composite fermions in graphene. *Phys. Rev. B* **2018**, *97*, 075113.

(12) Gouttenoire, V.; Barois, T.; Perisanu, S.; Leclercq, J. L.; Purcell, S. T.; Vincent, P.; Ayari, A. Digital and FM Demodulation of a Doubly Clamped Single-Walled Carbon-Nanotube Oscillator: Towards a Nanotube Cell Phone. *Small* **2010**, *6*, 1060–5.

(13) Laitinen, A.; Kaikkonen, J.-P.; Abhilash, T. S.; Todoshchenko, I.; Manninen, J.; Zavyalov, V.; Savin, A.; Isacsson, A.; Hakonen, P. J. A graphene resonator as an ultrasound detector for generalized Love waves in a polymer film with two level states. *Journal of Physics D: Applied Physics* **2019**, *52*, 24LT02.
